# Chronic Sinonasal Mucocele Manifesting as a Distinctive Collapsed Fibrous Hyalinized Cyst Wall

**DOI:** 10.1177/10668969261453368

**Published:** 2026-06-16

**Authors:** Liping Wang, Faizan Malik, Kumarasen Cooper

**Affiliations:** 1 Department of Pathology and Laboratory Medicine, Perelman School of Medicine, 6572 University of Pennsylvania, Philadelphia, PA, USA; 2 Department of Pathology and Laboratory Medicine, 12311 Penn State Health Hershey Medical Center, Pennsylvania State University College of Medicine, Hershey, PA, USA

**Keywords:** Chronic mucocele, hyalinized cyst wall, sinonasal

## Abstract

Chronic sinonasal mucoceles are benign expansile lesions caused by obstruction of sinus drainage, resulting in mucus accumulation and potential complications including nasal obstruction, facial pain, and visual disturbances. While their clinical aspects are well-characterized, detailed histopathologic descriptions remain limited. We conducted a retrospective clinicopathologic analysis of six patients treated for chronic sinonasal mucoceles between 2014 and 2024. Clinical data, imaging studies (computer tomography (CT) and magnetic resonance imaging (MRI)), endoscopic findings, and histopathologic features were reviewed. The cohort included four male patients and two female patients (age range: 31-85 years, mean: 61 years). Five patients presented with rhinologic symptoms; one was asymptomatic, with the lesion discovered incidentally. Anatomically, four muoceles (67%) involved the frontal-ethmoidal sinuses; the remaining two affected the sphenoid and maxillary sinuses, respectively. CT imaging revealed sinus opacification in five patients, with one showing marked mucosal and bony thickening. Histologically, all specimens exhibited collapsed fibrous walls with hyalinized stroma, sparse cellularity, and absent or denuded epithelium—finding consistent with late-stage changes from chronic pressure and reparative remodeling. Immunohistochemistry confirmed myofibroblastic differentiation of stroma cells. All patients underwent successful endoscopic management without recurrence. Chronic sinonasal mucoceles may present as pseudotumors with distinctive collapsed, hyalinized fibro-membranous walls, distinguishing them from early-stage lesions. Recognition of these characteristic histopathologic features, in conjunction with clinical and radiologic findings, is essential for accurate diagnosis and to avoid overtreatment. Endoscopic surgical management remains an effective treatment with excellent outcomes.

## Introduction

Chronic sinonasal mucocele is a benign, expansile, cyst-like lesion of the paranasal sinuses that results from obstruction of normal sinus drainage. Over time, retained mucus and secretions accumulate within the affected sinus, gradually expanding the cavity and causing bone remodeling or erosion due to pressure.^1^ Mucoceles most commonly arise in the frontal and ethmoid sinuses but may also occur in the maxillary or sphenoid sinuses.^2^ Clinically, they can remain asymptomatic for extended periods but may present with facial pain, headache, nasal obstruction, or even visual disturbances when expansion impinges on adjacent structures such as the orbit or cranial cavity. Chronic mucoceles are frequently associated with prior sinus surgery, trauma, chronic inflammation, or sinonasal tumors. Imaging, particularly computer tomography (CT) and magnetic resonance imaging (MRI), plays a crucial role in diagnosis,^1,3^ while treatment typically involves surgical drainage and re-establishment of sinus ventilation to prevent recurrence.^3^

Although clinical and surgical aspects of sinonasal mucoceles are well documented in the literature,^3^ only a limited number of case reports address their histopathologic features.^4–6^ Comprehensive descriptions remain scarce, which can pose diagnostic challenges when these lesions are encountered in routine practice. To help bridge this gap, we conducted a detailed radiographic and clinicopathologic analysis of a cohort of sinonasal mucoceles evaluated in our head and neck surgical pathology service.

## Materials and Methods

Six patients with chronic sinonasal mucocele were retrospectively identified between 2014 and 2024 in an academic institution with a large volume surgical center. For each patient, the following data were collected from the medical record: age, gender, anatomic location of the lesion, endoscopic findings, imaging studies including non-contrast CT sinus and MRI of the face or orbit, and surgical procedures performed. All available hematoxylin and eosin (H&E)-stained sections, and immunohistochemical stains were reviewed by two pathologists. This study was approved by the Institutional Review Board (IRB) committees of the hospital.

## Results

Our study included six patients comprising four male patients and two female patients with ages ranging from 31 to 85 years (mean age: 61 years, median age: 64 years). Clinically, five of six patients (Table 1) had a history of chronic sinusitis and presented with rhinologic symptoms, including rhinorrhea, nasal congestion, or facial pain or pressure. One patient was asymptomatic, with an incidental finding of a lesion in the left ethmoid sinus. The fronto-ethmoidal sinuses showed the highest frequency of involvement at 67% (4/6 patients). The remaining two patients had involvement of the sphenoid and maxillary sinus, respectively. Nasal endoscopy was performed in four patients before surgery, three of whom showed septal deviation; none demonstrated signs of acute inflammation or purulent discharge. Imaging studies revealed sinus opacification in five of six patients (Figure 1), while one lesion involving the sphenoid sinus showed pronounced mucosal and bony thickening (patient 3). MRI performed in 3 patients identified the relationship between mucocele and surrounding soft tissue and bone. Two patients with fronto-ethemoidal mucocele demonstrated endo-orbital extension; however, no endocranial extension was observed in any of them.

**Figure 1.**
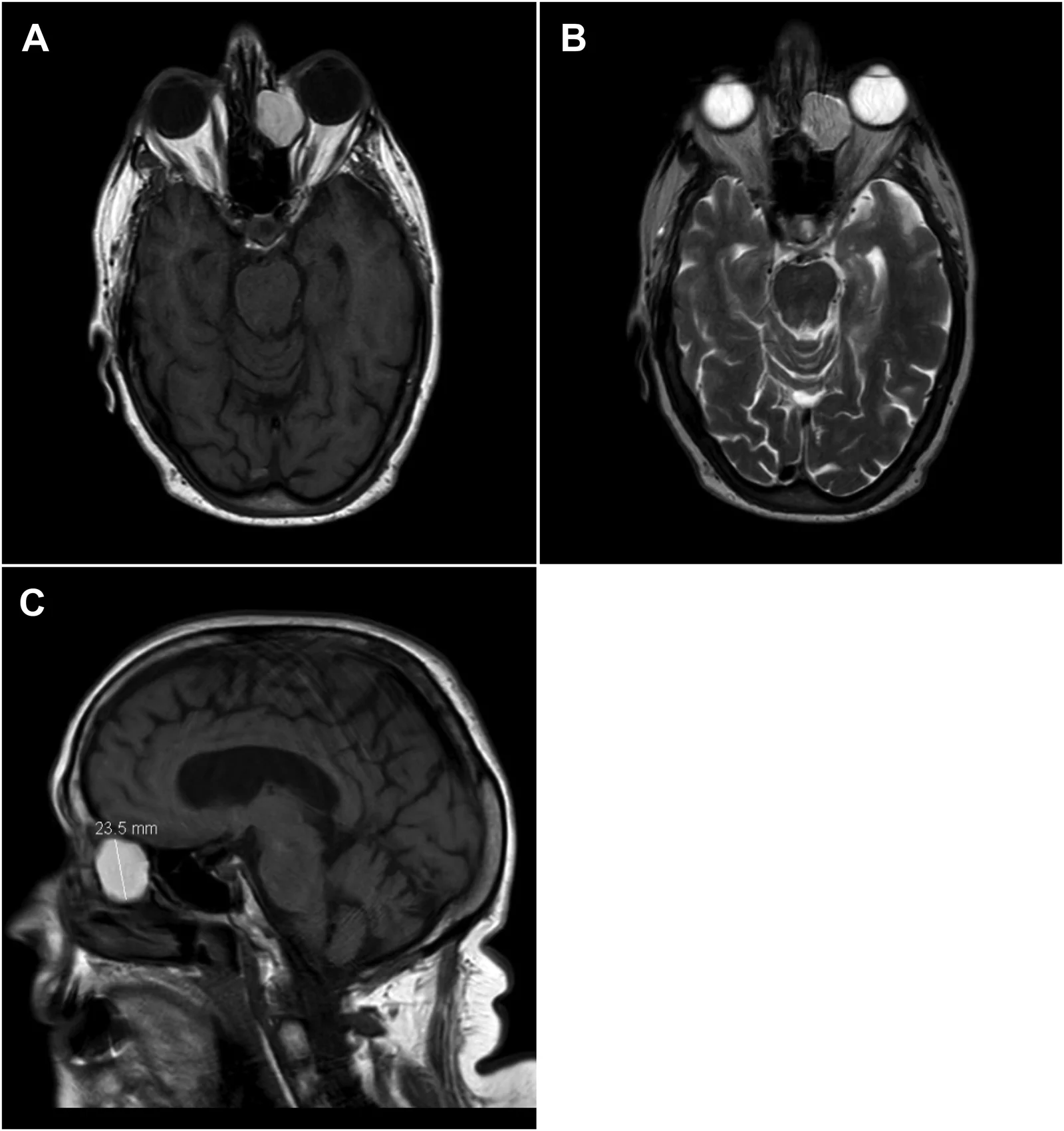
Preoperative Axial MR face imaging from patient #4, including T1-weighted (a), T2-weighted (b), along with sagittal T1-weighted imaging (c), show a 2.3 x1.7 x 2.8 cm lesion centered in the mid to anterior left ethmoid air cells with a portion bulging into the extraconal space to the left orbit. There was thinning of bone along the left ethmoid roof without intracranial extension. The superior aspect of opacification involved the left frontal sinus which was completely opacified.

**Table 1.** Clinicopathologic and Imaging Features of Our Cohort.

Patient# #	Age/Gender	Clinical History	Imaging Findings	Endoscopic Findings	Histological Findings
1	61 y/M	Rhinosinusitis symptoms with pressure and pain over nasal sinuses	CT: Retention cysts in b/l maxillary and sphenoid sinuses with small fluid levels; intact and deviated nasal septum and nasal bone spur in the left nasal cavity. Imaging findings suggest mild to moderate sinusitis.	Nasal septal deviation	Cyst wall with hyalinized stroma and respiratory epithelial lining
2	52 y/M	Rhinosinusitis symptoms	CT: Moderate to marked sinusitis involving the left frontal, b/l ethmoid, right sphenoid, and left maxillary sinuses	N/A	Cyst wall with hyalinized stroma, chronic inflammation, and respiratory epithelial lining;
3	31 y/M	Rhinosinusitis symptoms with pressure and pain over nasal sinuses	CT/MRI: Bone density lesion in sphenoid sinus, confluent with sphenoid bone and obscuring sphenoethmoidal recess, diagnostic considerations include exostosis, exuberant reactive bone thickening, or fibro-osseous lesion.	Nasal septal deviation and left inferior turbinate hypertrophy.	Multiloculated cystic lesion with hyalinized stroma, denuded respiratory epithelial lining
4	85 y/F	Single episode of vertigo with incidentally found left ethmoid sinus lesion, discovered on imaging studies; occupational history of long-term exposure to solvents	CT/MRI: Expansile opacification of the left ethmoid sinus with bone dehiscence along lateral margin; abutting the medial orbit but without intracranial extension. Imaging findings favor a mucocele.	b/l nasal septal deviation with large submucosal bulge with in the left middle meatus	Multiloculated cystic lesion with hyalinized stroma, denuded respiratory epithelial lining
5	67 y/F	Chronic sinusitis and previous FESS of frontal sinus	CT: Subcutaneous soft tissue mass communicating with the right frontal sinus, decreased in size, with persistent near complete opacification of the frontal sinuses, and multifocal osseous dehiscence in the medial orbital walls and orbital roofs. Possibly postsurgical change.	Scarring of bilateral lateral frontal sinuses recesses	Cyst wall fragments with hyalinization
6	72 y/M	Persistent bilateral nasal discharge and crusted secretions of both eyes; history of FESS of maxillary sinus	CT: b/l right maxillary sinus edema, Imaging findings favor chronic sinusitis	N/A	Cyst wall with hyalinized stroma, chronic inflammation, and respiratory epithelial lining;

M, male; F, female; b/l, bilateral; CT, computed tomography; MRI, magnetic resonance imaging; FESS, functional endoscopic sinus surgery; N/A, not available.

Histologically, all lesions exhibited uni- or multiloculated cystic structures with collapsed, fibrous, hyalinized cyst walls. On high-power examination, the cyst wall stroma demonstrated dense fibrosis and hyalinization with sparse cellularity, and minimal inflammation infiltrate (Figure 2). The mononuclear stromal fibroblasts/myofibroblasts showed regular nuclei without mitotic activity. Most lesions showed denuded or absent epithelial lining with only focal residual respiratory epithelium present. These features contrast with early-stage mucoceles, which typically show flattened, pseudostratified, ciliated, columnar epithelium and associated stromal inflammation. Special staining with Grocott's methenamine silver was negative for fungal organisms in one patient; periodic acid-Schiff (PAS) and trichrome stains highlighted the collagen component in the cyst walls, while mucicarmine confirmed increased mucus content within the cysts. Immunohistochemically, stromal cells were positive for smooth muscle actin (ACTA2) consistent with myofibroblastic differentiation, and negative for keratin AE1/AE3, STAT6, S100, SOX10, desmin (DES), EMA, MUC4, and β-catenin (CTNNB1).

**Figure 2.**
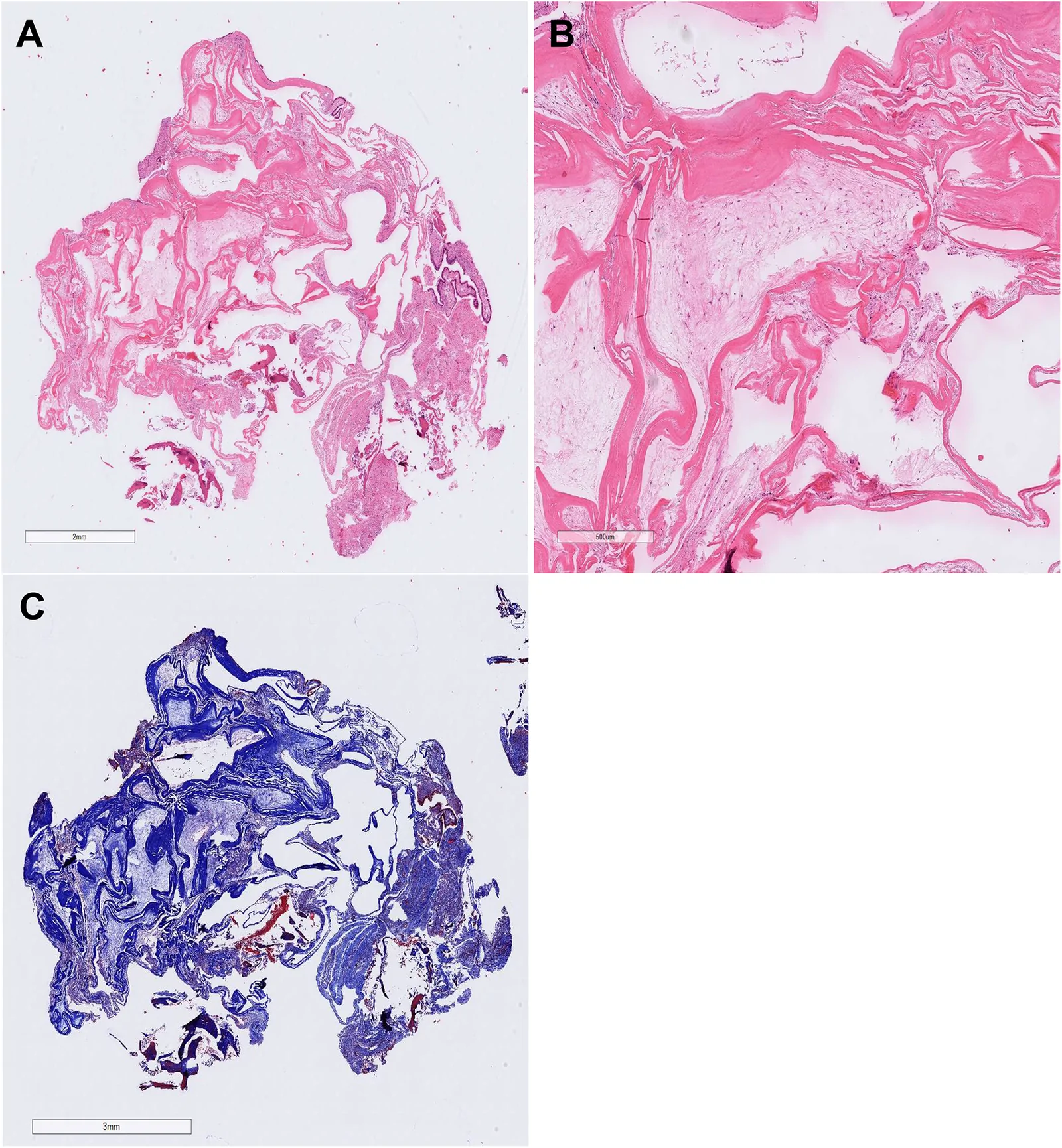
Chronic sinonasal mucocele show collapsed fibrous hyalinized cyst wall without epithelial lining (H&E stain, A and B), and trichrome stain highlight collagen (C).

## Discussion

Chronic sinonasal mucocele represents a benign but expansile lesion of the paranasal sinuses, resulting from obstruction of normal sinus drainage, leading to the accumulation of mucous secretions. Despite its non-neoplastic nature, it may present with clinically or radiologically aggressive features due to its mass effect on adjacent structures. This “pseudotumor” presentation can be misleading, making a thorough understanding of its pathologic features and radiologic appearance essential for accurate diagnosis, particularly to distinguish it from neoplastic processes.

Grossly, mucoceles present as well-circumscribed, cyst-like expansile lesions that remodel rather than invade surrounding bone. This distinction is crucial in the differential diagnosis with sinonasal malignancies.^1^ The contents of the mucocele are often sterile, viscous, and composed of mucin and desquamated epithelial debris. Secondary bacterial colonization can convert the mucocele into a mucopyocele,^7^ with a superimposed acute inflammatory component including neutrophilic infiltrates and suppurative material which were not observed in our cohort.

CT is the imaging modality of choice for initial evaluation. It typically reveals a homogenous, non-enhancing, expansile lesion that was also observed in our patients with smooth thinning or erosion of adjacent bony walls, particularly in the frontal or ethmoid sinuses. Unlike malignancies, mucoceles remodel bone rather than destroy it aggressively. The sinus walls may appear ballooned or scalloped, and there may be extension into adjacent compartments such as the orbit or anterior cranial fossa. In our series, endo-orbital extension was observed in two patients with fronto-ethemoidal mucocele, while no evidence of endocranial extension was noted in any of the patients.

MRI complements CT by providing better soft tissue characterization.^3^ On MRI, mucoceles are hypointense on T1-weighted and hyperintense on T2-weighted sequences in early stages, though signal intensity can vary based on protein content and viscosity of the mucoid material. These features were similarly present in our patients (Figure 1). Importantly, mucoceles do not enhance with contrast, except for the rim in inflamed or infected lesions. This helps distinguish them from enhancing neoplasms, such as inverted papillomas or sinonasal carcinomas, which show solid enhancement and often bony invasion.

Histologically, in the early stage, mucoceles are expanded by accumulated mucin and lined by pseudostratified columnar respiratory epithelium.^2,5^ In long-standing lesions, epithelial metaplasia, particularly squamous metaplasia, is commonly seen. The underlying stroma typically shows chronic inflammatory infiltrates, fibrosis, and occasionally cholesterol clefts and foreign body-type giant cells in response to extravasated mucin. However, in all six of our patients, the cyst walls were collapsed, fibrous, and hyalinized, with no identifiable epithelial lining present. These morphological features represent late-stage clustering of epithelial lining being flattened or denuded due to chronic pressure or cyst rupture and subsequent reparative changes, resulting in subsequent fibrous hyalinized cyst wall. The stromal myofibroblasts typically exhibit much lower cellularity compared to that seen in true neoplastic processes.

The primary diagnostic challenge of sinonasal mucoceles lies in differentiating them from infectious or neoplastic lesions. The differential diagnosis includes a variety of cystic and solid lesions that may affect the paranasal sinuses.^8^ Vascular lesions such as hemangiomas or vascular malformations may present with expansile growth and remodeling of adjacent bone, mimicking mucoceles, but are differentiated by the presence of endothelial cells lining the cystic spaces. Aneurysmal bone cysts of the head and neck region also come in the differential in the context of multicystic lesions but typically demonstrate blood filled spaces, lack of epithelial lining, and a characteristic radiographic appearance; rare these can arise from the sinus.^9,10^ Inflammatory myofibroblastic tumor (IMT) can occur in the head and neck^11^ and may be difficult to distinguish histologically, as they often contain chronic inflammatory cells, exhibit stromal hyalinization, and show ACTA2 positivity. However, IMT is typically more cellular and expresses ALK in most lesions, aiding in their identification. Low-grade fibromyxoid sarcoma can rarely present in the head and neck^12,13^ and may mimic sinonasal mucoceles, particularly when it exhibits low cellularity areas or cystically degeneration.^12^ In such lesions, differentiation from a mucocele can be challenging, as the diagnostic MUC4 immunohistochemical marker may also be expressed in mucus contents, potentially leading to diagnostic confusion. Among non-neoplastic entities, infectious cysts, including those caused by *Echinococcus* (hydatid disease), should also be considered, particularly in endemic regions.^14^ Histologically these cysts can be differentiated from mucoceles by the presence of germinal and laminated layers. Other entities in the differential include inflammatory polyps with cystic degeneration, antrochoanal polyp with reactive stromal atypia (absent in chronic sinonasal mucocele), respiratory epithelial adenomatoid hamartoma (REAH), and cholesterol granulomas. Accurate diagnosis requires integration of clinical history, imaging, and histopathologic features to distinguish mucoceles from these mimics.

We present the clinicopathologic and imaging features of a cohort of chronic sinonasal mucoceles, emphasizing their rare presentation as pseudotumors characterized by distinctive collapsed, hyalinized fibro-membranous wall—features that differ from those of early-stage lesions. Our findings indicate that endoscopic surgery serves both diagnostic and therapeutic purposes for most patients. Recognizing this entity as a neoplastic mimicker is crucial to avoid potential diagnostic pitfalls.

## References

[1] Allan Midyett F Mukherji SK . Skull base imaging: the essentials. 1st ed. Springer; 2020.

[2] Capra GG Carbone PN Mullin DP . Paranasal sinus mucocele. Head Neck Pathol. 2012;6(3):369-372. 10.1007/s12105-012-0359-2 PMC3422585 22623085

[3] Bouatay R Aouf L Hmida B , et al. The role of imaging in the management of sinonasal mucoceles. Pan Afr Med J. 2019;34:3. 10.11604/pamj.2019.34.3.18677 PMC6850736 31762872

[4] Magboul NA Alzubaidi AA Abumsmar LA Alzarei A Al-Ahmari M Alshehri MA . Mucocele of the paranasal sinuses: retrospective analysis of a series of eight cases. Cureus. 2023;15(7):e41986. 10.7759/cureus.41986 PMC10427889 37593272

[5] Thompson LD . Paranasal sinus mucocele. Ear Nose Throat J. 2012;91(7):276-278. 22829031

[6] Ting MYL Shan M Gantz O Zhang-Nunes S Wrobel B . Optic neuropathy due to an ethmoid mucocele: a case report’and literature review. Case Rep Ophthalmol. 2019;10(2):227-234. 10.1159/000501898 PMC6760354 31692625

[7] Kshar A Patil A Umarji H Kadam S . Mucopyocele of’the’maxillary sinus. Dent Res J (Isfahan). 2014;11(1):119-123. PMC3955306 24688571

[8] Ozturk K Gawande R Gencturk M Boegel K Caicedo-Granados E Cayci Z . Imaging features of sinonasal tumors on positron emission tomography and magnetic resonance imaging including diffusion weighted imaging: a pictorial review. Clin Imaging. 2018;51:217-228. 10.1016/j.clinimag.2018.05.018 29879597

[9] McMullen PD Bridge JA Blair EA Yang CW Collins J Cipriani NA . Aneurysmal bone cyst of the maxillary sinus’with USP6 rearrangement: case report of a rare entity and review of the literature. Head Neck Pathol. 2019;13(3):281-285. 10.1007/s12105-018-0956-9 PMC6684671 30076509

[10] Han Y Yang X Tang L , et al. Primary aneurysmal bone’cyst’of nasal cavity and sinuses in a child: case report and literature review. Ear Nose Throat J. 2023;102(4):231-238. 10.1177/01455613221081569 35428416

[11] Kerr DA Thompson LDR Tafe LJ , et al. Clinicopathologic and genomic characterization of inflammatory myofibroblastic tumors of the head and neck: highlighting a novel fusion and potential diagnostic pitfall. Am J Surg Pathol. 2021;45(12):1707-1719. 10.1097/PAS.0000000000001735 34001695

[12] He KF Jia J Zhao YF . Low-grade fibromyxoid sarcoma with cystic appearance and osseous metaplasia in the cheek: a case report and review of the literature. J Oral Maxillofac Surg. 2013;71(6):1143-1150. 10.1016/j.joms.2012.12.017 23499160

[13] Gjorgova Gjeorgjievski S Fritchie K Thangaiah JJ Folpe AL Din NU . Head and neck low-grade fibromyxoid sarcoma: a clinicopathologic study of 15 cases. Head Neck Pathol. 2022;16(2):434-443. 10.1007/s12105-021-01380-y PMC9187798 34559373

[14] Bouckaert MM Raubenheimer EJ Jacobs FJ . Maxillofacial hydatid cysts. Oral Surg Oral Med Oral Pathol Oral Radiol Endod. 2000;89(3):338-342. 10.1016/s1079-2104(00)70099-0 10710460

